# MG63 Osteoblast-Like Cells Exhibit Different Behavior when Grown on Electrospun Collagen Matrix versus Electrospun Gelatin Matrix

**DOI:** 10.1371/journal.pone.0031200

**Published:** 2012-02-02

**Authors:** Shiao-Wen Tsai, Hau-Min Liou, Cheng-Jie Lin, Ko-Liang Kuo, Yi-Sheng Hung, Ru-Chun Weng, Fu-Yin Hsu

**Affiliations:** 1 Institute of Biochemical and Biomedical Engineering, Chang-Gung University, Tao-Yuan, Taiwan; 2 Institute of Bioscience and Biotechnology, National Taiwan Ocean University, Keelung, Taiwan; 3 Department of Life Sciences, National Taiwan Ocean University, Keelung, Taiwan,; University of California, Merced, United States of America

## Abstract

Electrospinning is a simple and efficient method of fabricating a non-woven polymeric nanofiber matrix. However, using fluorinated alcohols as a solvent for the electrospinning of proteins often results in protein denaturation. TEM and circular dichroism analysis indicated a massive loss of triple-helical collagen from an electrospun collagen (EC) matrix, and the random coils were similar to those found in gelatin. Nevertheless, from mechanical testing we found the Young's modulus and ultimate tensile stresses of EC matrices were significantly higher than electrospun gelatin (EG) matrices because matrix stiffness can affect many cell behaviors such as cell adhesion, proliferation and differentiation. We hypothesize that the difference of matrix stiffness between EC and EG will affect intracellular signaling through the mechano-transducers Rho kinase (ROCK) and focal adhesion kinase (FAK) and subsequently regulates the osteogenic phenotype of MG63 osteoblast-like cells. From the results, we found there was no significant difference between the EC and EG matrices with respect to either cell attachment or proliferation rate. However, the gene expression levels of OPN, type I collagen, ALP, and OCN were significantly higher in MG63 osteoblast-like cells grown on the EC than in those grown on the EG. In addition, the phosphorylation levels of Y397-FAK, ERK1/2, BSP, and OPN proteins, as well as ALP activity, were also higher on the EC than on the EG. We further inhibited ROCK activation with Y27632 during differentiation to investigate its effects on matrix-mediated osteogenic differentiation. Results showed the extent of mineralization was decreased with inhibition after induction. Moreover, there is no significant difference between EC and EG. From the results of the protein levels of phosphorylated Y397-FAK, ERK1/2, BSP and OPN, ALP activity and mineral deposition, we speculate that the mechanism that influences the osteogenic differentiation of MG63 osteoblast-like cells on EC and EG is matrix stiffness and via ROCK-FAK-ERK1/2.

## Introduction

Controlling cell behavior is of critical importance for tissue engineering, regenerative medicine, and the study of cellular molecules. To date, considerable efforts have been made to develop scaffolds for tissue engineering. The ideal scaffold should be both biodegradable and bioactive, and it should mimic the structure and biological function of the native extracellular matrix as much as possible in terms of both chemical composition and physical structure. The native extracellular matrix contains structural protein fibrils such as collagen and elastin that range from tens of nanometers to micrometers in scale. These nanofibrils entangle with each other and form an organized structural matrix that guides tissue morphogenesis and remodeling in vivo. In addition, the matrix fibrils also serve as a reservoir for growth factors and cytokines that regulate cell migration, proliferation, and differentiation [1].

Researchers as early as Weiss introduced the concept of contact guidance to describe cell orientation and cell locomotion in response to underlying substrata [2]. Cells attach to, and organize around, fibers with diameters smaller than those of the cell. In recent decades, it has become widely accepted that cellular activities such as adhesion, spreading, migration, and proliferation are sensitive to the molecular composition and chemical properties of the substrate surface. Moreover, the physical properties and nanotopography of the matrix are capable of affecting cellular behaviors such as adhesion, morphology, migration, gene expression, surface antigen display, and cytoskeletal function [3], [4]. Collagen is the most commonly used biomaterial in matrix construction for tissue engineering, and it is known to affect the growth and differentiation of a variety of cell types. Individual triple-helix molecules of collagen undergo self-assembly to form fibrils with a characteristic D-pattern in vivo or in vitro under well-controlled conditions. Gelatin is derived from acid or alkaline hydrolysis of native collagens. The amino acid compositions of collagen and gelatin are almost identical; however, other characteristics such as secondary structure, isoelectric point, and molecular weight distribution are different. Hypothesizing that the nanotopographic features of designed substrates are important for modulating cellular behavior, we previously showed that varying the nanotopography of a collagen matrix with and without the D-period affects the behavior of osteoblasts [5]. We found that the unique D-pattern of collagen not only enhances the mineralization process of osteoblasts but also induces the cells to display their normal phenotype.

Electrospinning technology is one of the popular techniques being used to manufacture nanofibrous scaffolds from biological and/or synthetic polymers, and it has tremendous potential for biomedical applications, including wound dressings [6], drug delivery carriers [7] and tissue-engineering scaffolds [8]. Collagen is the most commonly used biomaterial in matrix construction for tissue engineering, and it is known to affect the growth and differentiation of a variety of cell types.

Fluorinated alcohols such as 2, 2, 2-trifluoroethanol (TFE) and 1, 1, 1, 3, 3, 3-hexafluoroisopropanol (HFIP) are good solvents for polypeptide biopolymers such as collagen and gelatin. Unfortunately, most studies of electrospinning using either pure collagen or hybrid collagen with other polymers often lead to protein denaturation as a result of electrospinning highly volatile organic solvents such as HFIP and TFE [9], [10]. Hence, denaturation of collagen takes place and the matrix structure is similar to that of gelatin at the molecular scale. Since gelatin is derived from hydrolysis of collagen, generally speaking “denatured collagen”. Zeugolis [11] suggests that the electrospinning of pure collagen nanofibers is an expensive way to make gelatin.

Although both chemical and biological stimulation are known to influence the behavior of cultured cells, physical stimulation also plays a key role in preserving the native behavior of cells during in vitro cultivation. In particular, mechanical stimulation has been reported to play an important role in modulating chondrocyte metabolism and, in turn, cartilage homeostasis [12]. Numerous studies have demonstrated that chondrocytes secrete more GAGs under tensile stimulation compared with static culture [13], [14]. Along with the extra stress of stimulation, the stiffness of the culture substrate is another source of physical stimulation for attached cells [15]. Therefore, we hypothesize difference of matrix stiffness between electrospun collagen matrix and gelatin matrix will affect intracellular signaling through the mechano-transducers Rho kinase (ROCK) and focal adhesion kinase (FAK) and subsequently regulates the osteogenic phenotype of MG63 osteoblast-like cells. In the present work, we examined the attachment, morphology, proliferation and differentiation of MG-63 osteoblast-like cells on electrospun collagen and gelatin matrices to evaluate whether using electrospun collagen as a cell matrix is worthwhile, even though at the molecular scale an EC matrix is almost the same as an EG matrix.

## Materials and Methods

### Reagents

Modified Eagle's medium (MEM), fetal bovine serum (FBS), and trypsin were purchased from GIBCO. Gelatin Type A was purchased from Sigma-Aldrich Chemical Company. The other chemicals used were of reagent grade unless otherwise stated. Type I collagen was prepared from calf skin, as previously described [16], and acid-soluble lyophilized type I collagen was used for all experiments.

### Preparation of electrospun collagen and gelatin matrices

Collagen and gelatin powder were dissolved in 1,1,1,3,3,3-hexafluoro-2- propanol at a concentration of 8% (w/v). For the process of electrospinning, the polymer solution was placed into a 5 ml syringe fitted with a needle with a tip diameter of 0.96 mm and attached to a syringe pump that provided a steady solution flow rate of 0.508 ml/hr. Electrospinning voltage was applied to the needle at 23 kV using a high-voltage power supply, and the tip-to-collector distance was fixed at 10 cm. The polymer solution formed a Taylor cone upon exit and was collected on 15 mm diameter glass coverslips placed on a grounded collector in the form of nonwoven nanofibers.

### Characterization of electrospun nanofibers

The fiber morphologies of the electrospun collagen matrix (EC) and the electrospun gelatin matrix (EG) were examined by scanning electron microscopy (Hitachi S-4800). Briefly, the electrospun matrices were sputter-coated with gold and visualized by scanning electron microscopy at an accelerating voltage of 15 kV. The scanning electron microscopic images were analyzed with Image-Pro Express (Media Cybernetics) to determine the average fiber diameter. The average fiber diameter and standard deviation were calculated from 100 random measurements.

### Circular dichroism analysis

Samples (1 mg) were dissolved in 1 ml acetic acid (0.5 M) or HFIP. Circular dichroism spectra of samples were analyzed on a Jasco Model J-810 spectropolarimeter (Jasco, UK) using a quartz cylindrical cuvette with a path length of 0.1 mm. The cuvette was filled with 50 µl of sample for each measurement. Circular dichroism spectra were obtained by continuous wavelength scans from 215 to 235 nm at a scan-speed of 200 nm/min.

### Mechanical testing

The electrospun matrix were cross-linked prior to mechanical testing with dry state. The mechanical properties of the EC and EG, approximately 10 mm×20 mm×0.1 mm (width×length×thickness), were measured by a uniaxial testing machine (Tinius Olsen H1KT) with a 5-N load cell under a cross-head speed of 1 mm/min (n = 10). The Young's modulus (E) and ultimate tensile strength (UTS) of the sheets were measured in static mode.

### Cell culture on EC and EG

Both EC and EG readily dissolved in aqueous media, necessitating that they be crosslinked before cellular incubation could begin. The electrospun matrix was cross-linked by treatment with N-(3-dimethylaminopropyl)-N-ethylcarbodiimide hydrochloride (EDC). The matrix was immersed in 5 mM EDC solution in 95% ethanol for 24 h at room temperature. The cross-linked matrix was immersed in 75% ethanol for 24 h and then washed repeatedly with 0.01 M PBS to remove the residual EDC. Finally, the matrix was washed with DMEM and was UV sterilized for 16 h.

### Cellular attachment

MG63 osteoblast-like cell line was purchased from BCRC (Bioresource Collection and Research Center, Taiwan). The electrospun matrices were placed into 24-well tissue culture dishes containing a suspension of MG63 osteoblast-like cells (BCRC no. 60279) (5×10^4^ cells/well) in MEM supplemented with 2 mM L-glutamine, ascorbic acid (50 µg/ml), 10 mM ß-glycerophosphate, 0.1 mM non-essential amino acid, 1 mM sodium pyruvate, 100 U/mL penicillin and 100 µg/mL streptomycin and 10% fetal bovin serum and incubated at 37°C in a humidified atmosphere of 5% CO_2_. The cultures of cell-seeded electrospun matrices were harvested at 1, 2, 3 and 4 h.

### Cellular proliferation

The electrospun matrices were placed into 24-well tissue culture dishes containing a suspension of MG63 osteoblast-like cells (5×10^4^ cells/well) in MEM supplemented with 2 mM L-glutamine, ascorbic acid (50 µg/ml), 10 mM ß-glycerophosphate, 0.1 mM non-essential amino acid, 1 mM sodium pyruvate, 100 U/mL penicillin and 100 µg/mL streptomycin and 10% fetal bovin serum and incubated at 37°C in a humidified atmosphere of 5% CO_2_. The culture medium was changed once every three days. The cultures of cell-seeded electrospun matrices were harvested at 2 h and on days 2, 4, 7 and 14.

### WST-1 assay kit

Cell attachment and proliferation was measured using a WST-1 assay kit (Clontech). The WST-1 assay used to determine cell viability is based on the reductive cleavage of tetrazolium salt to the soluble formazan by a mitochondrial dehydrogenase that is active only in viable cells. The amount of dye formed was immediately measured by a microplate reader (Biotek uQuant) at a wavelength of 450 nm. At each time point, four samples were used to measure the number of cells attached to the electrospun matrices. The experiments were performed in triplicate.

### Fluorescent staining of the cytoskeleton

Cellular morphology was investigated by examining the F-actin cytoskeleton fluorescently stained with fluorescein isothiocyanate (FITC)-conjugated phalloidin and the nucleus stained with DAPI. After being cultured for 4 h, the cells were fixed with 3.7% paraformaldehyde in phosphate buffer for 10 min before being washed twice with 0.02 M PBS (pH 7.4). The cells were then rinsed in PBS containing 0.1% Triton X-100 for 5 min. To reduce nonspecific background staining, the samples were blocked with 1% bovine serum albumin (BSA) in PBS for 1 h. After blocking, the BSA solution was aspirated and the samples were incubated with 6.4 µM FITC-conjugated phalloidin for 20 min. The cells were then incubated with DAPI solution for 3 min to stain the DNA in the cells. After three 5 min washes with PBS, the samples were observed under a laser scanning confocal microscope (LSCM, Zeiss LSM 510 META).

### Alkaline phosphatase activity

Alkaline phosphatase activity was assayed according to the method described by Lowry et al. [17] In brief, to lyse cells the matrices were washed with PBS and then suspended in 0.5 mL PBS containing 0.1 M glycine, 1 mM MgCl_2_ and 0.05% Triton X-100. Following lysis, 100 µl of the lysate was incubated with 250 µl of p-nitrophenyl phosphate solution at 37°C for 30 min. Enzymatic activity was terminated by adding 100 µl of ice cold 3 M NaOH, and the amount of *p*-nitrophenol liberated was measured by monitoring light absorbance at 405 nm. The amount of *p*-nitrophenol should be equivalent to alkaline phosphatase activity. The results of the alkaline phosphatase activity assay were normalized by the viability of the cells on the matrix.

### Gene expression analysis using real-time PCR

#### RNA extraction

After 7, 14, or 21 days of culturing, total RNA was extracted from the samples using TriReagent according to the manufacturer's instructions. In brief, ice-cold Tri Reagent (1 mL) was added to the samples, and they were incubated for 5 min at room temperature. Then, 1-bromo-3-chloropropane (BCP; 0.1 mL) was added, and they were incubated for a further 15 min at room temperature. The samples were centrifuged at 12,000× g for 15 min at 4°C, and the upper aqueous phase that contained the RNA was collected. The RNA was precipitated using isopropanol and centrifuged at 12,000× g for 10 min at 4°C. Following precipitation, 1 mL of 75% ethanol was added into the RNA pellet, followed by centrifugation at 7500× g for 5 min, and the resulting pellet was air dried. The RNA pellet was dissolved in DEPC water. The concentration and purity of each sample were assessed by absorbance at 260 nm and by the 260/280 nm ratio, respectively.

### Quantitative real-time PCR

Real-time PCR was used to determine the levels of the osteopontin (OPN), osteocalcin (OCN), collagen type I, and β-actin mRNA transcripts and of 18S ribosomal RNA. Table 1 shows the sequences of the oligonucleotides that were used as PCR primers. Briefly, the reaction volume (25 µL) included 12.5 µL SYBR Green PCR Master Mix (Protech SA-SQGLR-V2), 3 µL diluted cDNA (15 ng), 0.5 µL of MgCl_2_ (25 mM), 4 µL of ddH_2_O and 2.5 µL each of forward and reverse primers (10 µM). After initial denaturation at 94°C for 15 min, the target genes were amplified with 40 cycles of denaturation at 94°C for 15 s and annealing at 62.5°C for 60 s. Real-time PCR reactions were carried out by an iQ5 Gradient Real Time PCR system (Bio-Rad). The levels of RNA expression were determined according to the 2^−ΔΔCt^ method. The expression levels of the target genes were calculated by normalizing the mRNA level for a particular gene against that of 18S ribosomal RNA as an internal control. The fold changes were calculated using the following formulas:
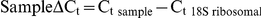


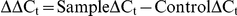


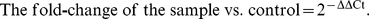



**Table 1:** Oligonucleotide primer for PCR amplification.

Gene	Primer sequence: sense/antisense
Collagen Type I	5′-CGGAGGAGAGTCAGGAAG-3′
	5′-CAGCAACACAGTTACACAAG-3′
Osteopontin	5′-AAGCGAGGAGTTGAATGG-3′
	5′-CTCATTGCTCTCATCATTGG-3′
Osteocalcin	5′-CAGCGAGGTAGTGAAGAGAC-3′
	5′-GCCAACTCGTCACAGTCC-3′
β-actin	5′-GGAACGGTGAAGGTGACAG-3′
	5′-TGGACTTGGGAGAGGACT-3′
18S ribosomal RNA	5′-GAAGATATGCTCATGTGGTGTTG-3′
	5′-GTCTTAGGTGCGGTCATGTTC-3′

### Immunoblotting analysis

Cells were seeded on substrates at 3×10^4^ cells/cm^2^ in medium. Immunoblotting of focal adhesion kinase (FAK), phosphorylated-focal adhesion kinase (p-FAK) and extracellular signal-regulated kinase (ERK) was performed after 2 h of cell seeding. Immunoblotting of osteoblast differentiated-specific proteins was performed after day 14 and day 21 of culture. Proteins were collected in distilled water supplemented with 1% (v/v) Triton X-100, 1 mM MgCl_2_, 20 mM Tris, 150 mM NaCl, 1 mM Tris-EDTA, 10% glycerol, 1 mM DTT, 1 mM PMSF, 1 X protein inhibit, 10 mM β-glycerophosphate, 1 mM NaF, 0.1 mM Na_3_VO_4_, 0.1% SDS and 1% NP40. The cell supernatants were collected after centrifugation for 10 min at 15,000× g at 4°C. Protein concentration was determined with a Bradford Coomassie assay. Proteins were fractionated by electrophoresis and electrotransferred to polyvinylidene difluoride film (PVDF). Blocking was performed using 5% (w/v) non-fat milk/PBST (0.2%, PBS with 0.02% (v/v) Tween 20) at 4°C overnight. The primary antibody was diluted with fresh blocking buffer to the designated concentration and applied to the membrane at 4°C overnight. Antibodies specific to FAK (Santa Cruz Biotechnology-INC.sc-558), p-FAK (Santa Cruz Biotechnology-INC. sc-11765-R), ERK1/2 (Millipore, 06-182), bone sialoprotein (Millipore AB1854), nucleophosmin (Invitrogen 325200), OPN (Millipore AB1870) and β-actin (Millipore AB1501) were used. After reacting with a secondary antibody, immunoreactive bands were visualized using enhanced chemiluminescence detection (Millipore WBKLS0500). The electrophoretic bands were analyzed by an image analysis program (ImageJ software 1.42, National Institutes of Health, USA). Nucleophosmin B23 was used as an internal control.

### Alizarin Red-S staining for mineralization

The formation of calcium phosphate by MG63 osteoblast-like cells was determined as described by using the alizarin red-S assay [18]. The medium was removed, and the cell layers on the matrix were rinsed with PBS 3 times and fixed in 3.6% (v/v) formaldehyde at room temperature for 15 min. The fixed cells were stained with 2% Alizarin red S (pH 4.1–4.3) for 15 min at 25°C. Then, the cell layers were washed with deionized water and observed by microscopy (Nikon TS-100). For quantification of staining [19], 800 µL of 10% (v/v) acetic acid was added to each matrix, and the matrix was incubated at room temperature for 30 min with shaking. The cell layers on the matrix were scraped and transferred to a 1.5-mL microcentrifuge tube. After vortexing for 30 s, the slurry was overlaid with 500 µL mineral oil, heated to 85°C for 10 min, and transferred to ice for 5 min. The slurry was centrifuged at 20,000× g for 15 min and 500 µL of the supernatant was removed to a new 1.5-mL microcentrifuge tube. Then, 200 µL of 10% (v/v) ammonium hydroxide was added to neutralize the acid. The absorbance of the supernatant was measured in triplicate at 405 nm.

### Statistical analyses

Statistical analyses were performed using SPSS v.10. For each condition, at least 100 fibers were chosen randomly to measure fiber diameter. Fiber diameters were analyzed with Student's t test. Cellular viability, alkaline phosphatase activity, gene expression, immunoblotting analysis and mineralization assay were analyzed with the non-parametric Mann-Whitney U test. Differences at *p*<0.05 were considered statistically significant.

## Results

### Characterization of EC and EG

**Figure 1:**
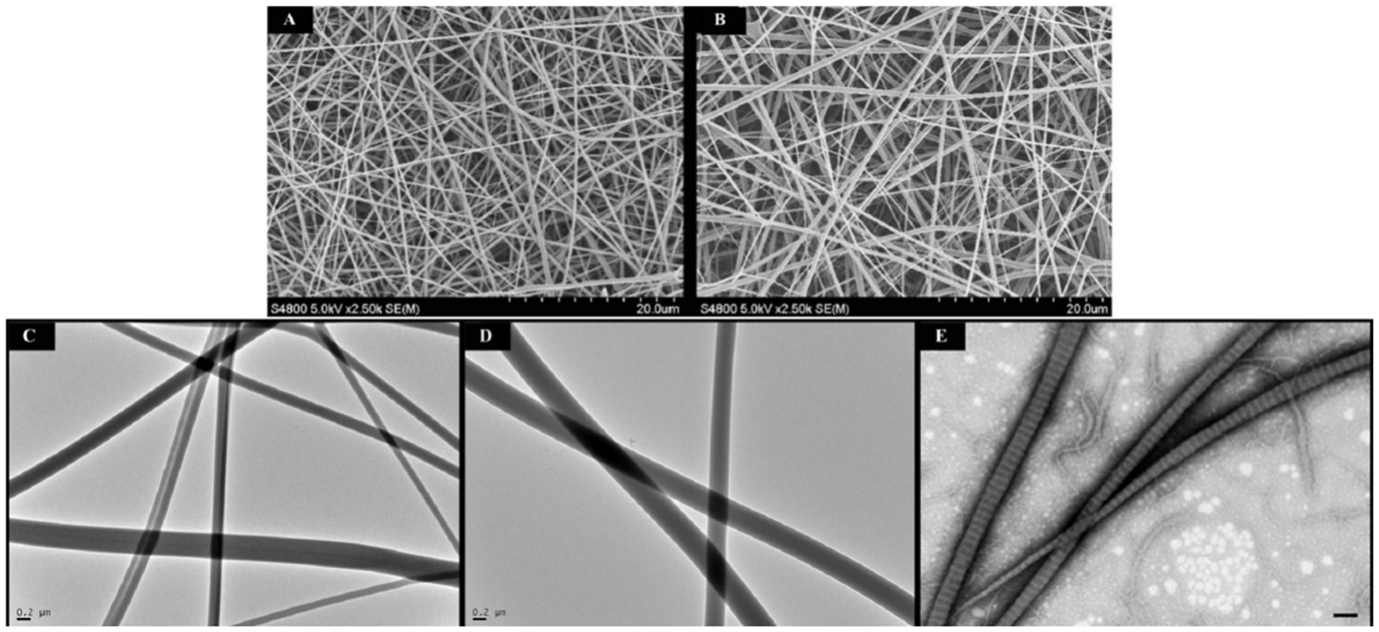
Electron microscopic images of electrospun collagen matrix (EC) and electrospun gelatin matrix (EG). Scanning electron microscopic images of (A) electrospun collagen matrix (EC), and (B) electrospun gelatin matrix (EG) at 2,500× magnification. The EC nanofibers had an average diameter of 692±214 nm, and the EG nanofibers had an average diameter of 573±368 nm. Scale bar is 20 µm. Transmission electron microscopic images of (C) electrospun collagen matrix, (D) electrospun gelatin matrix and (E) self-assembled collagen matrix. Scale bar is 0.2 µm. EC nanofibers did not exhibit the characteristic D-periods pattern that was apparent for the native collagen molecules self-assembled into collagen fibrils. Self-assembled collagen was formed by dialyzed collagen solution (in acetic acid) against 0.02 M phosphate buffered-saline (PBS, pH 7) at 4°C. Fibril formation (self-assembled) was initiated by warming the mixture to 37°C for 4 hours.

**Figure 2:**
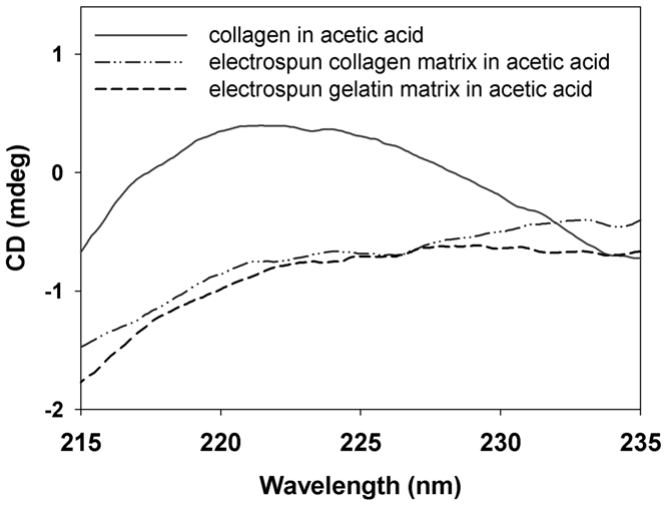
CD spectra of the different matrix preparations. Circular dichroism spectra of the acid-solubilized materials plotted as mean residue ellipticity (mdeg×cm^2^×dmol^−1^)] vs. wavelength (nm). The triple-helical structure of native collagen have a characteristic positive peak at around 220 nm. The EC curve indicated a massive loss of triple-helical structure and similar to EG.

Figure 1A and B shows SEM micrographs of the EC and EG under 2500× magnification. Image analysis indicated that the collagen nanofibers had an average diameter of 692±214 nm, and the gelatin nanofibers had an average diameter of 573±368 nm. There was no significant difference between the diameters of collagen and gelatin nanofibers (*p*>0.05). However, the EC nanofibers did not exhibit the characteristic cross-striation pattern that was apparent for the self-assembled collagen fibers (Figure 1C and 1E). To gain information on the triple-helical secondary structure of the EC and EG, circular dichroism measurements were performed. The circular dichroism spectra of native collagen, EC and EG in acetic acid were measured and are shown in Figure 2. The EC exhibited a circular dichroism spectrum indicating a massive loss of triple-helical collagen and suggesting random coils similar to those seen in EG.

**Figure 3:**
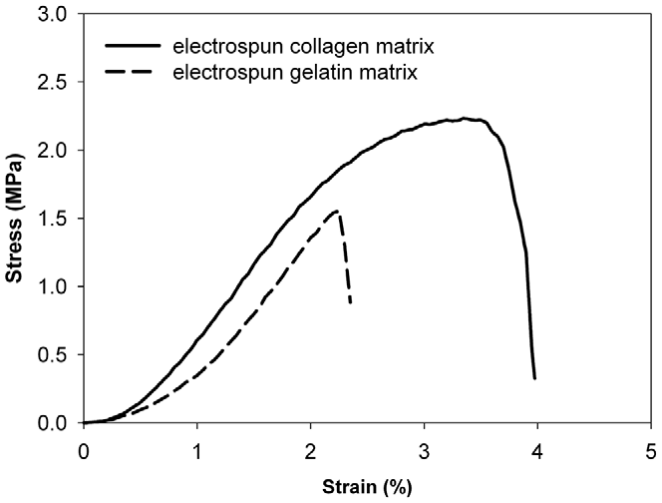
The tensile stress-strain curve of an electrospun collagen matrix and an electrospun gelatin matrix. The Young's modulus of the EC and EG were 94.29±15.18 and 71.88±21.10 MPa, respectively. The ultimate tensile stresses the EC and EG were 1.93±0.37 and 0.93±0.46 MPa, respectively.

When the EC and EG were strained uniaxially, a “J-shaped” stress-strain curve was observed (shown as in Figure 3). The Young's modulus of the EC and EG were 94.29±15.18 and 71.88±21.10 MPa, respectively, they were determined from a linear regression of the linear region of the stress-strain curve. The ultimate tensile stresses the EC and EG were 1.93±0.37 and 0.93±0.46 MPa, respectively. The above results indicate that the mechanical strength of EC is greater than that of EG (*p*<0.05).

### Cellular attachment and proliferation

**Figure 4:**
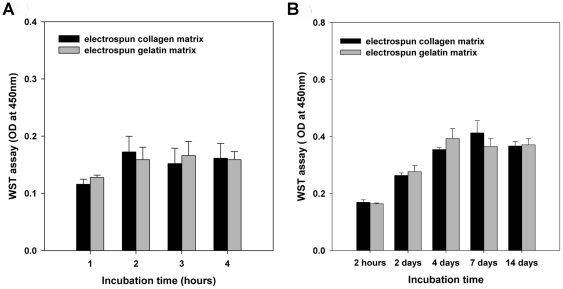
Quantification of MG63 osteoblast-like cells on EC and EG matrices. WST assay quantifying cell attachment and proliferation on electrospun scaffolds of collagen and gelatin. (A) The attachment of MG63 osteoblast-like cells on various matrices after culturing for up to 4 hours. (B) The viability of MG63 osteoblast-like cells on various matrices after culturing for up to14 days. Data are presented as mean ± SD, n = 4. Statistical analysis was compared between EC and EG. There were no significant differences in cellular attachment or proliferation between EC and EG (*p*>0.05).

**Figure 5:**
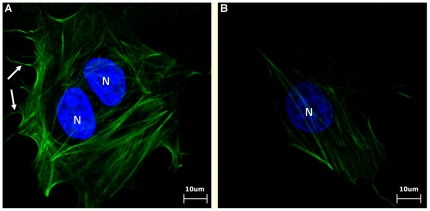
Different cellular morphology of cells on EC and EG matrices. Fluorescent microscopic micrographs of MG63 osteoblast-like cells cultured on matrix for 4 hours. (A) electrospun collagen matrix. (B) electrospun gelatin matrix. Cells cultured on EC showed well-organized F-actin stress fibers. On the other hand, cells cultured on EG showed with numerous, very thin and long filopodia. Cytoskeletal F-actin is stained green with FITC and cell nuclei are stained blue with DAPI. (white arrow: filopodia, Scale bar = 10 µm)

The attachment and proliferation of MG63 osteoblast-like cells on the EC and EG nanofibrous matrices were quantified using WST-1 assays. There were no significant differences in cellular attachment or proliferation between EC and EG (*p*>0.05) (as shown in Figure 4). To test the effects on cell morphology, we incubated the MG63 osteoblast-like cells on EC and EG. Cells that adhered to the matrix were examined by staining their actin cytoskeleton with FITC-phalloidin and visualizing them with a laser scanning confocal microscope. Most of the cells adhered to either matrix after a 4 h attachment period appeared to be spread (as shown in Figure 5). However, cells cultured on EC showed well-organized F-actin stress fibers. Moreover, numerous, very thin and long filopodia could also be observed on the cells grown on EC.

### Alkaline phosphatase activity

**Figure 6:**
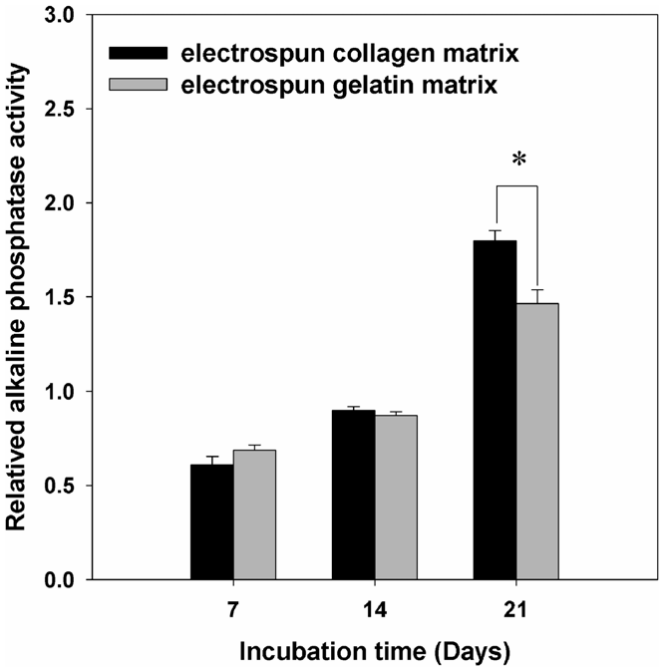
Analysis of alkaline phosphatase activity of cells on EC and EG matrices. The alkaline phosphatase activity of cells was found to be significantly higher on EC than on EG on day 21. Data are presented as mean ± SD, n = 4. Statistical analysis was compared between EC and EG. (*) denotes a significant difference (*p*<0.05).

Alkaline phosphatase activity is recognized as an early marker for the activity of osteoblasts. The phenotype of the cells cultured on the nanofibrous matrices was evaluated by measuring their alkaline phosphatase activity after culturing for up to 21 days, as shown in Figure 6. The alkaline phosphatase activity of MG63 osteoblast-like cells was found to be significantly higher on EC than on EG on day 21 (*p*<0.05).

### Real-time PCR analysis

**Figure 7:**
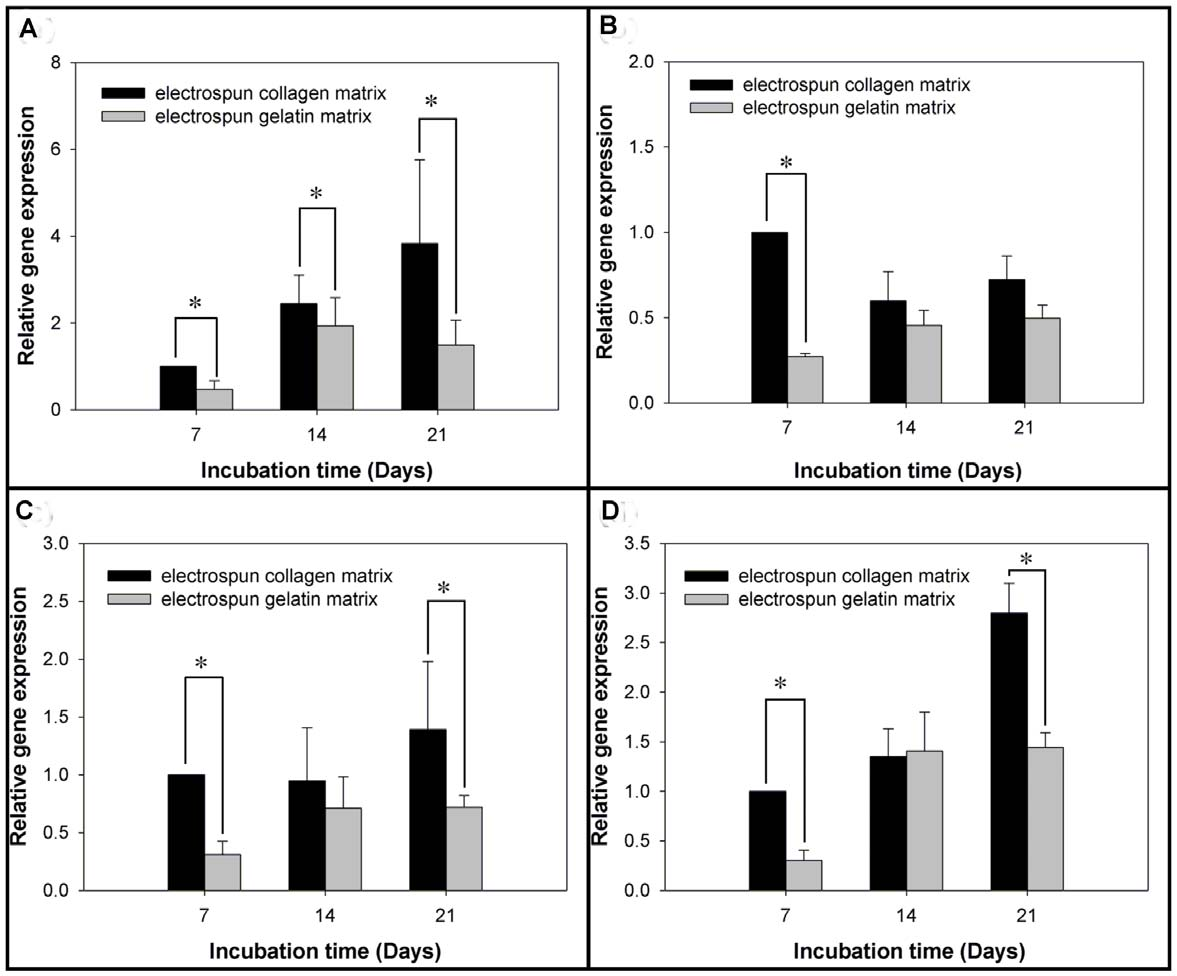
Matrices regulate the bone-associated genes expressed by MG63 osteoblast-like cells. Real–time PCR analyses of bone-associated genes expressed by MG63 osteoblast-like cells on various matrix. (A) β-actin levels, (B) type I collagen levels, (C) OPN levels and (D) OCN levels after normalization to 18S ribosomal RNA levels. Data are shown as the fold change relative to the electrospun collagen matrix after 7 days of incubation. On day 7, the cells showed significantly higher expression levels of β-actin, type I collagen, OPN and OCN when grown on EC. And, the expression levels of β-actin, OPN and OCN were significantly higher on day 21 when grown on EC. Data are presented as mean ± SD, n = 4. Statistical analysis was compared between EC and EG. (*) denotes a significant difference (*p*<0.05).

The expression levels of MG63 osteoblast-specific genes were analyzed using QPCR for type I collagen, OCN, OPN, β-actin and 18S ribosomal RNA, as shown in Figure 7. On day 7 (the earlier time point), the MG63 osteoblast-like cells showed significantly higher expression levels of β-actin, typeI collagen, OCN and OPN when grown on EC (*p*<0.05). Furthermore, the expression levels of β-actin, OPN and OCN were significantly higher on day 21 when grown on EC (*p*<0.05).

### Western blot analysis

**Figure 8:**
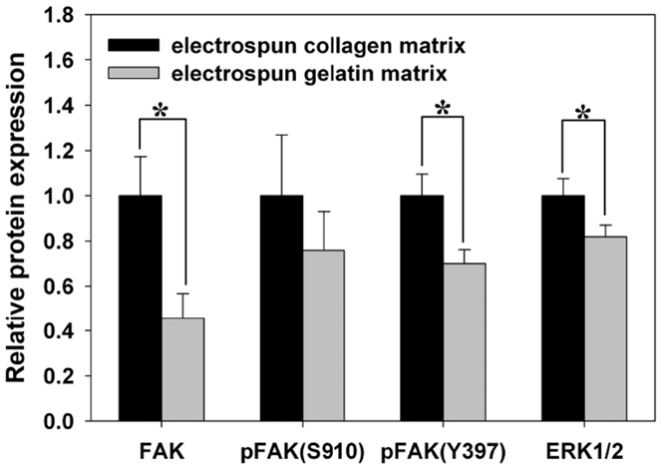
Effects of matrix on FAK and phospho-FAK and ERK1/2 activation in MG63 osteoblasts-like cell. Densitometric quantitation of the protein bands was performed after normalizing to nucleophosmin B23 levels. (Figure S1 shown the images of full western blots). Data are shown as the fold change of the corresponding 2 h of incubation on the EC. The total FAK levels expressed by MG63 osteoblast-like cells were significant higher in cells grown on EC than in those grown on EG. Moreover, the level of FAK phosphorylation at tyrosine 397 was lower in cells grown on EG than in those grown on EC. Data are presented as mean ± SD, n = 3. Statistical analysis was compared between EC and EG. (*) denotes a significant difference (*p*<0.05).

**Figure 9:**
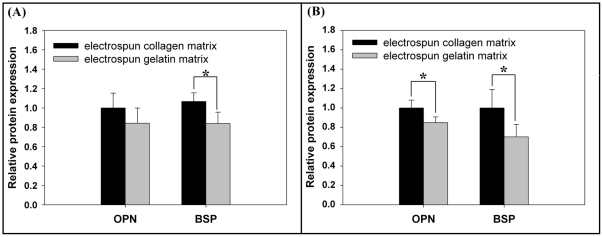
Effects of matrix on BSP and OPN protein expression in MG63 osteoblasts-like cell. Western blot analysis for BSP and OPN proteins in cells cultured for (A) 14 and (B) 21 days. Densitometric quantitation of the OPN and BSP protein band were performed after normalizing to nucleophosmin B23 levels (Figure S2 shown the images of full western blots). Data are shown as the fold change of the corresponding on the electrospun collagen matrix. The expression level of BSP and OPN proteins showed a significant increase in cells grown on EC at 21 days. Data represent mean ± SD, n = 3. Statistical analysis was compared between EC and EG. (*) denotes a significant difference (*p*<0.05).

The total FAK levels expressed by MG63 osteoblast-like cells were significant higher in cells grown on EC than in those grown on EG (*p*<0.05). Moreover, the level of FAK phosphorylation at tyrosine 397 was lower in cells grown on EG than in those grown on EC (*p*<0.05) (Figure 8 and Figure S1). The expression levels of MG63 osteoblast-specific proteins were also analyzed, as shown in Figure 9 and Figure S2. In comparison to cells grown on EG, the expression level of BSP protein showed a significant increase in cells grown on EC at 14 and 21 days (*p*<0.05). The MG63 osteoblasts also showed significantly higher expression levels of OPN when grown on EC at day 21 (*p*<0.05).

### Mineralization activity

**Figure 10:**
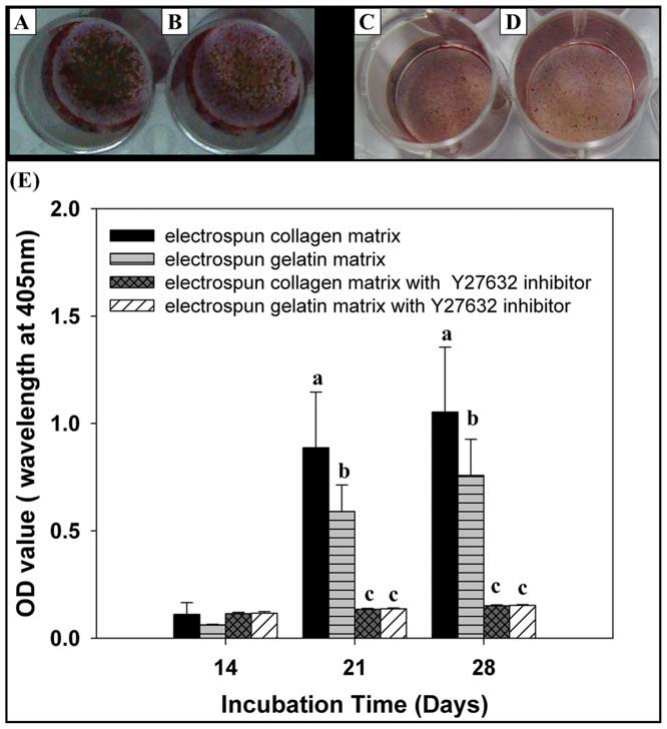
Effects of matrix on bone mineralization in MG63 osteoblasts-like cell. Optical images of ARS staining for mineralization in MG63 osteoblast-like cells for day 28 (A) EC, (B) EG. (C) EC with 10 µM Y27632, (D) EG with 10 µM Y27632. Quantification of mineral deposition by Alizarin Red-S staining. Data represent mean ± SD, n = 4. Statistical analysis was compared between EC and EG with or without Y27632. Different letters represent significance at *p*<0.05 by non-parametric Mann-Whitney U test.

The deposition of calcium on EC or EG by MG63 osteoblasts was quantitatively determined by colorimetric calcium quantification (Figure 10). It was evident that the mineralization was time-dependent. Significantly higher calcium content was always observed in EC throughout the entire experiment. In order to prove the mechanism that influences the osteogenic differentiation of MG63 osteoblast-like cells on EC and EG is via ROCK-FAK-ERK1/2. We further inhibited ROCK activation with a ROCK-specific inhibitor Y27632 at 10 µM during differentiation to investigate the effects on matrix-mediated osteogenic differentiation. Results showed the extent of mineralization was decreased with inhibition after induction by 3 and 4 weeks. Moreover, it is no significantly difference between EC and EG. (*p*>0.05)

## Discussion

A porous and nanofibrous biodegradable matrix that mimics natural matrix can provide an optimal scaffold for tissue engineering [20], [21], [22]. EC and EG have been widely used to fabricate scaffolds that serve as active analogues of the native extracellular matrix. Type-A gelatin is a water-soluble protein derived from collagen with acid. But collagen is an optically active protein that adopts the polyproline II-like helical conformation with a weak positive maximum absorption band at 210–230 nm in circular dichroism spectrum analysis [23]. Gelatin, the denatured form of collagen, is characterized by degraded α-chains and a lack of internal structure or configurational order [24]. The different molecular structures of the two proteins resulted in various cellular biological responses when they used as cell-cultured matrices.

Xu has shown that there are stronger interactions between cells and fibers when the fiber diameter is smaller than the size of the cells [25]. Consequently, most researchers believe that a nanofibrous matrix that has a high surface area-to-volume ratio and that a well defined architecture may promote cell growth more effectively than a sponge substrate [26], [27]. Electrospinning is a convenient approach for generating nanofibers. Currently, the most widely adopted method for the electrospinning of either pure collagen or gelatin utilizes highly volatile fluoroalcohols such as HFIP or TFE. However, fluorinated alcohol is used to denature proteins due to its strong interaction with both polar and non-polar amino acid side groups. In our past research, images of collagen fibrils in FE-SEM showed features characteristic of native collagen fibers such as a rope-like structure and a regular banding pattern. However, we found that electrospun collagen nanofibers lack this rope-like structure and regular banding pattern [8]. The electrospun collagen nanofibers did not exhibit the characteristic cross-striation pattern of native collagen, and the circular dichroism spectrum of acid-solubilized, electrospun collagen nanofibers lacked the positive peak, similarly to the gelatin preparation. The above results indicate that the electrospinning of collagen out of fluoroalcohols results in the formation of denatured collagen that is similar to gelatin at the molecular scale. The finding seems suggested that there is no worthy to make effors to develop EC since most properties are similar with EG. As well-known cell-materials interactions were not only depended on chemical properties but also on the physical properties.

As known, the higher polymer molecular weight or narrower molecular weight distribution has higher mechanical strength [28]. Collagen molecule is composed of individual triple helix (2 α1 and 1α2 chains) with invariant molecular weight around 290 KDa. On the contrary, gelatin is derived from hydrolysis of native collagens that means gelatin is a single polypeptide chain with lower molecular weight and broader molecular weight distribution compared collagen. (as shown in Figure S3). The higher molecular weight and narrower molecular weight distribution resulted in EC has higher mechanical strength than EG. However, inferior differences in gelatin purity cannot be excluded.

The mechanical properties of the EC and EG were measured by a uniaxial testing machine. A similar “J-shaped” stress-strain curve reflects the typical stress-strain behavior of collagen-based tissues and fibers. There are three major regions of the stress-strain curve: 1) the toe region, 2) the linear region and 3) the yield and failure region. The “toe region” was the zone between zero strain and the intersection of the slope of the linear region of stress-strain curve on the horizontal axis (strain) [29]. Tensile testing was performing to analyze the mechanical properties of collagen-based matrix has been reported extensively. The stress-strain curves of those studies indicated that the mechanical property of collagen-based matrix is depending on many factors such as animal species, extraction method and crosslink density et al [30], [31]. In the toe region of tress-train curve of tendon and ligament, that should be to reflect the uncrimping of the collagen fibrils in the fiber, as well as the initiation of stretching of the triple helix, nonhelical ends and crosslink [32]. Xu pointed out that the initial compliant “toe region” is due to the network orientation changes toward the loading axis on collagen scaffold [33]. Hence, we speculated the lower stress might cause the crimped nanofibers in the EC or EG to stretch out in the toe region.

In the work, we would like to find out the physical property how to influence the cellular responses. Cellular activities such as attachment, spreading, migration, proliferation, differentiation and maturation are sensitive to the surface topography, molecular composition, and mechanical properties of the matrix. Hanagata [34] compared the effects of two different type I collagen structures (a network structure of fibrils and a feltwork structure of filamentous molecules) on osteoblast-like cells and found that the proliferation and differentiation of these cells were delayed on the fibrillar collagen. Tsai studied the effects of three types of collagen structures (acid soluble, fibrillar and heat-denatured) on osteoblast-like cell behavior. They found that heat-denatured collagen promoted the osteoblasts to enter the differentiation stage [35]. Taubenberger [36] found that partially-denatured collagen exposes RGD-motifs and stimulates osteoblast adhesion, spreading, motility and differentiation. However, we observed no significant differences in cell attachment, morphology or proliferation between cells grown on EC and cells grown on EG.

Bone formation depends on osteoblasts, which synthesize and secrete bone-specific extracellular matrix proteins such as osteocalcin, osteopontin, type I collagen and bone sialoprotein (BSP), and which are responsible for matrix deposition and mineralization [37]. Osteopontin (OPN), a major component of the bone matrix, is expressed at different stages of bone formation [38]. Type I collagen is expressed throughout bone formation process. BSP is a highly post-translationally-modified acidic phosphoprotein that is normally expressed in mineralized tissues such as bone and dentin [39]. The precise function of BSP is still unknown; however, several factors strongly suggest that BSP may have a multifaceted role in the development of mineralized tissue. Gordon demonstrated that increased BSP expression, by itself, has a direct effect on osteoblast differentiation, as shown by increased Runx2/Cbfa-1 activity and mineralized nodule formation [40]. OCN, known as the bone Gla protein, is a vitamin K- and D-dependent protein that is expressed by highly differentiated osteoblasts during the mineralization stage [41]. The OCN gene expression of osteoblasts was found to be upregulated through collagen binding to integrins after type I collagen secretion [42]. In our study, we found that MG63 osteoblast-like cells grown on EC exhibited superior characteristics of maturation compared to those grown on EG, as indicated by the expression of osteogenic genes such as OPN, type I collagen and OCN.

Ratanavaraporn [43] found that collagen scaffolds had significantly higher compressive modulus levels than gelatin scaffolds. In addition, he found no significant differences in initial cell attachment or cell proliferation between cells grown on gelatin scaffolds and cells grown on collagen scaffolds. Evans proved that cell attachment was unaffected by the stiffness of the growth substrate, but that osteogenic differentiation of stem cells increases as a function of substrate stiffness [44]. Mechanotransduction plays a key role in the biological processes of a variety of cell types, including stem cells, smooth muscle cells, endothelial cells, and bone cells. Cells can sense and respond to the mechanical properties of their environment. Cell response to substrate stiffness has been suggested to play an important role in tissue engineering.

Focal adhesion kinase (FAK) is localized to focal adhesions and is believed to activate multiple signaling pathways that mediate cell attachment, proliferation, and differentiation. For instance, FAK induces RhoA to stimulate actomyosin contraction [45] and proliferation [46], and it induces Rac1 to regulate lamellipodia formation [47]. FAK signaling regulates mechanotransduction by responding to substrate rigidity [48]. Kocgozlu [49] investigated how matrix stiffness affects FAK-Y397 auto-phosphorylation. Takeuchi [50] showed that the attachment of MC3T3-E1 cells to collagen causes phosphorylation of FAK, which in turn activates MAPK, and that this signaling cascade is critical for osteoblastic differentiation. Khatiwala [51] has previously shown that enhanced FAK phosphorylation leads to higher RhoA activity on stiffer matrices, thus promoting increased cellular contractility through ROCK. The extracellular signal-regulated kinase (ERK) has previously been demonstrated to be a key regulator for growth, differentiation, integrin expression, and cell function in human osteoblastic cells [52]. Xiao proved that integrin-ECM interactions activate osteoblast-specific gene expression through the MEK/ERK pathway and result in increased cbfa1/Runx2 activity [53]. Khatiwala also demonstrated that cross-talk between RhoA-ROCK and the ERK-MAPK pathways can stimulate phosphorylation of ERK through MAPK to regulate the activity of Runx2 and drive differentiation toward a mature osteoblastic phenotype. Moreover, FAK Ser910 phosphorylation is mediated through an ERK-dependent pathway in response to environmental stimuli [54]. Based on these reports, we examined the protein expression levels of FAK, phosphorylated FAK, ERK1/2, OPN and BSP by western blotting, alkaline phosphatase activity, and mineralization assays to evaluate the influence of EC and EG stiffness on MG63 osteoblast-like cell differentiation. Not surprisingly, we found that the protein levels of phosphorylated Y397-FAK, phosphorylated S910-FAK, ERK1/2, BSP and OPN, as well as ALP activity, were all modulated by matrix stiffness. Staining the mineral deposition of cells with ARS dye after 21 days of culture, as in Figure 10, showed that the mineralization of osteoblasts was significantly greater when they were grown on a collagen matrix as opposed to gelatin. Based on these results, we speculate that the mechanism that influences the osteogenic differentiation of MG63 osteoblast-like cells on EC and EG is matrix stiffness.

### Conclusion

Although most of the characteristics of EC are similar to those of EG, one difference is matrix stiffness. This physical property resulted in higher mRNA levels of OPN, type I collagen, ALP, and OCN; higher protein levels of phosphorylated Y397-FAK, phosphorylated S910-FAK, ERK1/2, BSP and OPN; and higher ALP activity in cells grown on EC than in cells grown on EG. These findings suggest that the different matrix stiffness of EC compared with EG modulated cellular responses even though most of their chemical properties and topography are the same. Moreover, it is still a worthwhile endeavor to develop an EC that preserves the typical biological properties of collagen as a scaffold for tissue engineering.

## Supporting Information

Figure S1 **The image of western blot for FAK, p-FAK at tyrosine 397 (Y397) and serine 910 (S910), ERK1/2 and nucleophosmin B23 proteins in cells cultured for 2 hours. M: marker.**
(TIF)

Figure S2 **The image of western blot for BSP, OPN and nucleophosmin B23 proteins in cells cultured for (A) 14 and (B) 21 days. M: marker.**
(TIF)

Figure S3 **The image of SDS-Page for collagen (lane C), gelatin (lane G), electrospun collagen (lane EC) and electrospun gelatin (lane EG). M: marker.**
(TIF)
