# Supplementary figures and images for: MG63 Osteoblast-Like Cells Exhibit Different Behavior when Grown on Electrospun Collagen Matrix versus Electrospun Gelatin Matrix
Source: PLoS One. 2012 Feb 2;7(2):e31200. doi: 10.1371/journal.pone.0031200 (PMC3271086; doi:10.1371/journal.pone.0031200)

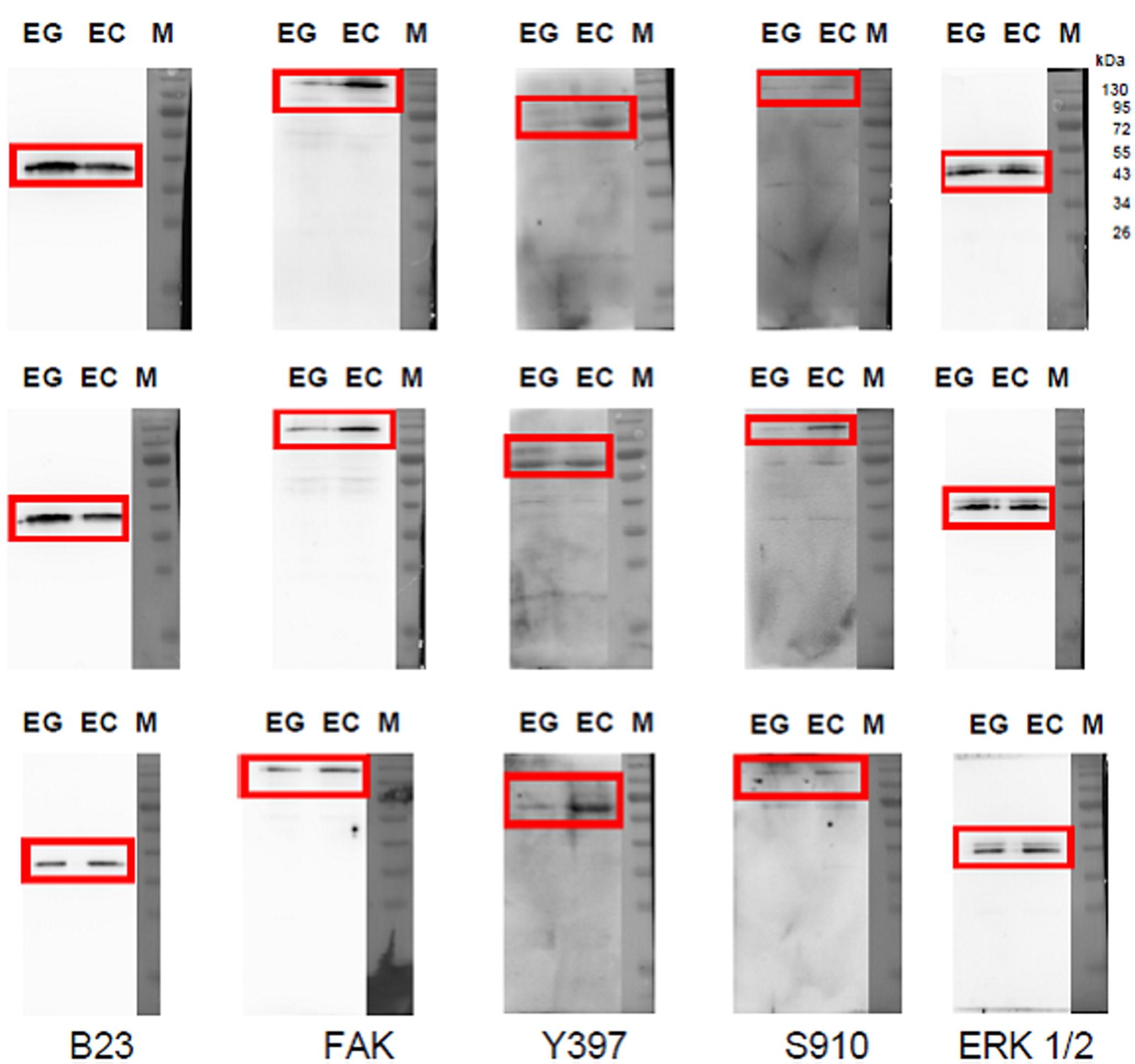

Supplement: Figure S1 — The image of western blot for FAK, p-FAK at tyrosine 397 (Y397) and serine 910 (S910), ERK1/2 and nucleophosmin B23 proteins in cells cultured for 2 hours. M: marker. (TIF) [file pone.0031200.s001.tif]

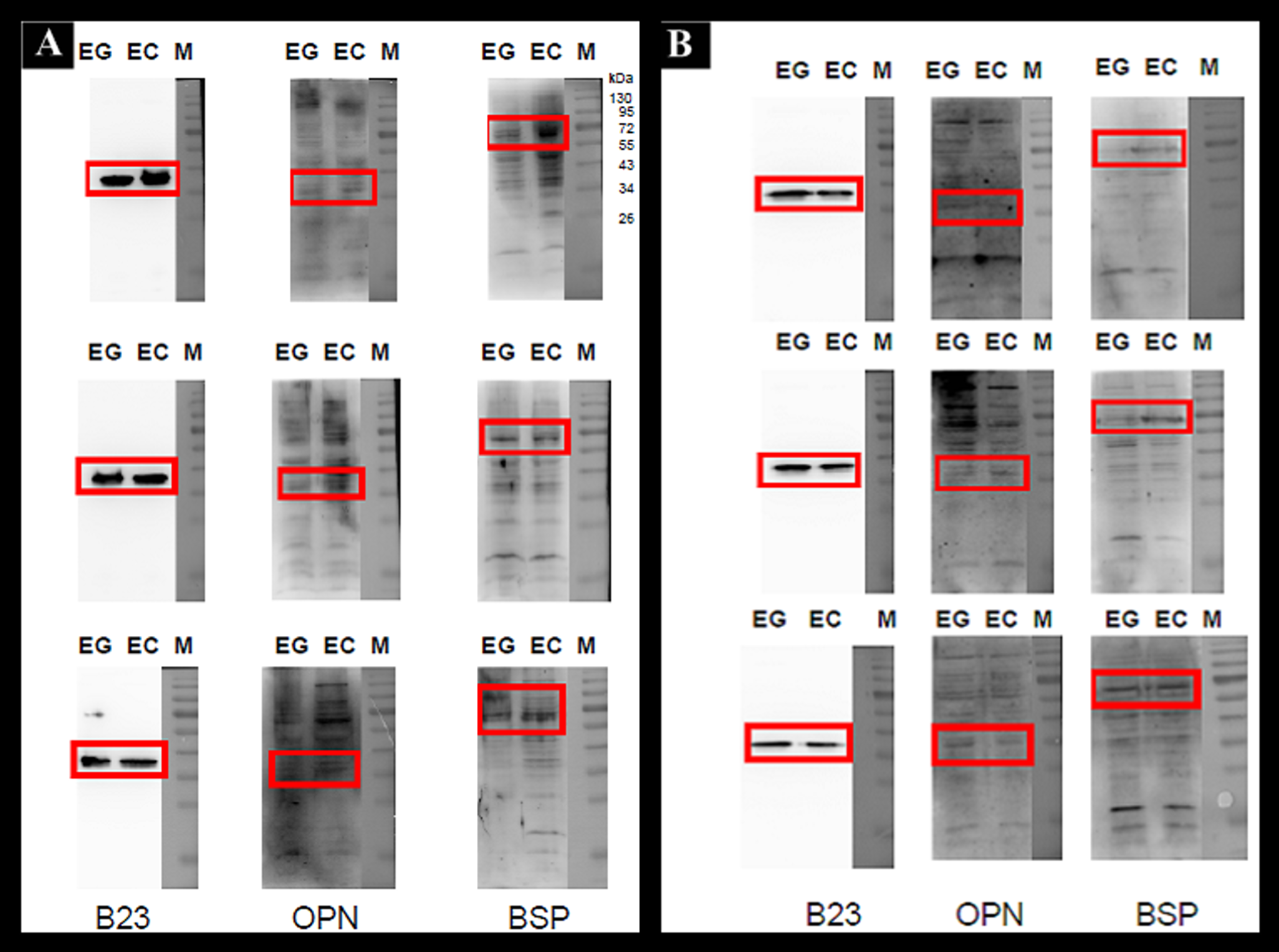

Supplement: Figure S2 — The image of western blot for BSP, OPN and nucleophosmin B23 proteins in cells cultured for (A) 14 and (B) 21 days. M: marker. (TIF) [file pone.0031200.s002.tif]

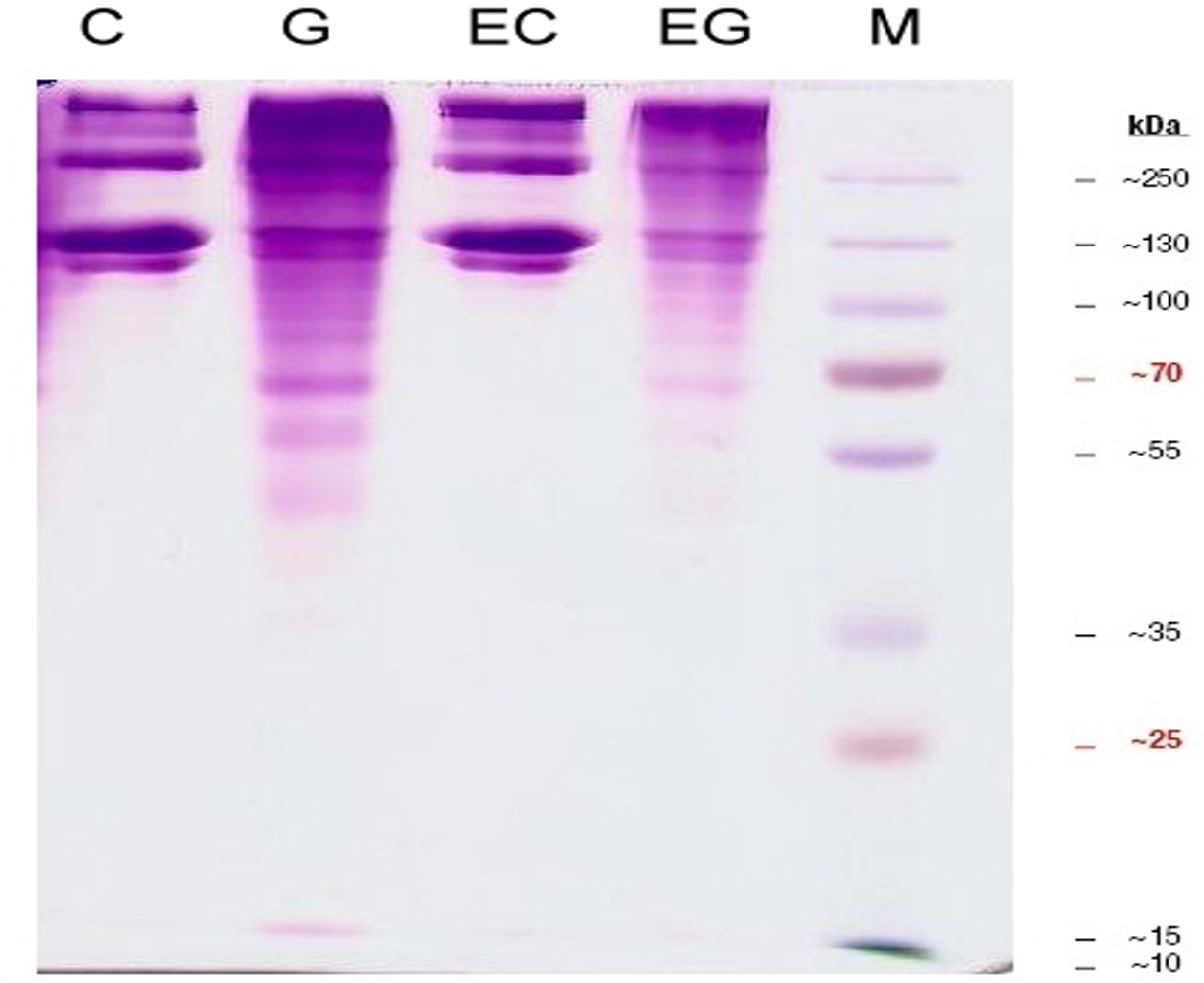

Supplement: Figure S3 — The image of SDS-Page for collagen (lane C), gelatin (lane G), electrospun collagen (lane EC) and electrospun gelatin (lane EG). M: marker. (TIF) [file pone.0031200.s003.tif]
